# Supplementary material for: Ongoing Exposure to Peritoneal Dialysis Fluid Alters Resident Peritoneal Macrophage Phenotype and Activation Propensity
Source: Front Immunol. 2021 Jul 27;12:715209. doi: 10.3389/fimmu.2021.715209 (PMC8353194; doi:10.3389/fimmu.2021.715209)
Supplement: Supplementary file 1 [file DataSheet_1.pdf]

Supplementary Figure S1

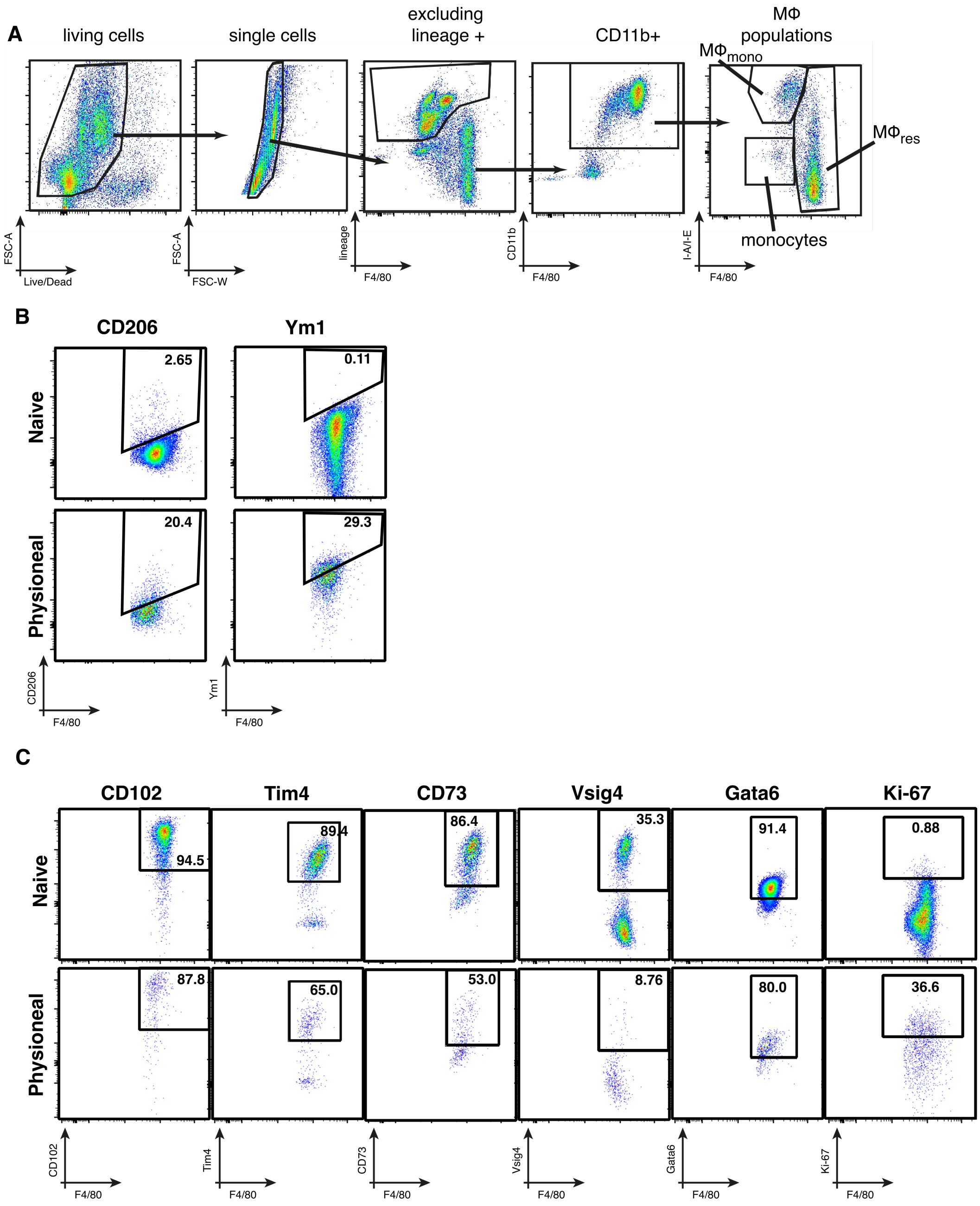

Figure S1: (A) Gating strategy employed to identify MΦres, MΦmono and Ly6C high monocytes. (B) Example gating of MΦres for expression of CD206 and Ym1 6 h after a single Physioneal injection. (C) Example gating to identify expression of CD102, Tim4, CD73, Vsig4, Gata6 and Ki-67 in MΦres 24 h after 9 Physioneal injections. Placement of gates in (B) & (C) was based on appropriate FMO controls.

Supplementary Figure S2

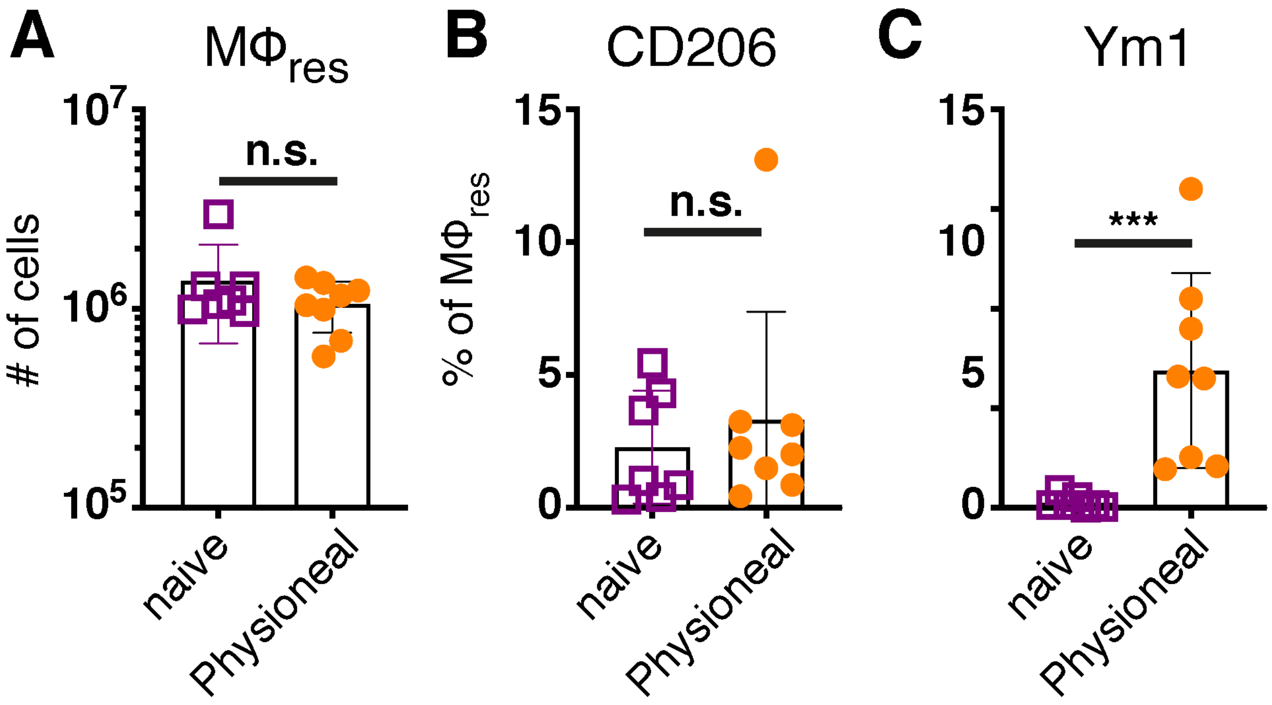

Figure S2: The effects of PD fluid injection on peritoneal MΦres are transient.

C57BL/6 mice were injected with Physioneal (orange circle) or left untreated (purple square) and whole PEC isolated 24 h p.i.. Cells were analysed by flow cytometry to determine the number of MΦres (A) expression of cellular activation markers (B & C).

Datapoints depict individual animals and bars indicate mean and SD. Data pooled from 2 independent experiments using 3-4 animals per group. Data analysed using a Mann-Whitney-U test after transformation. n.s.: not significant; ***: $p<0.001$

Supplementary Figure S3

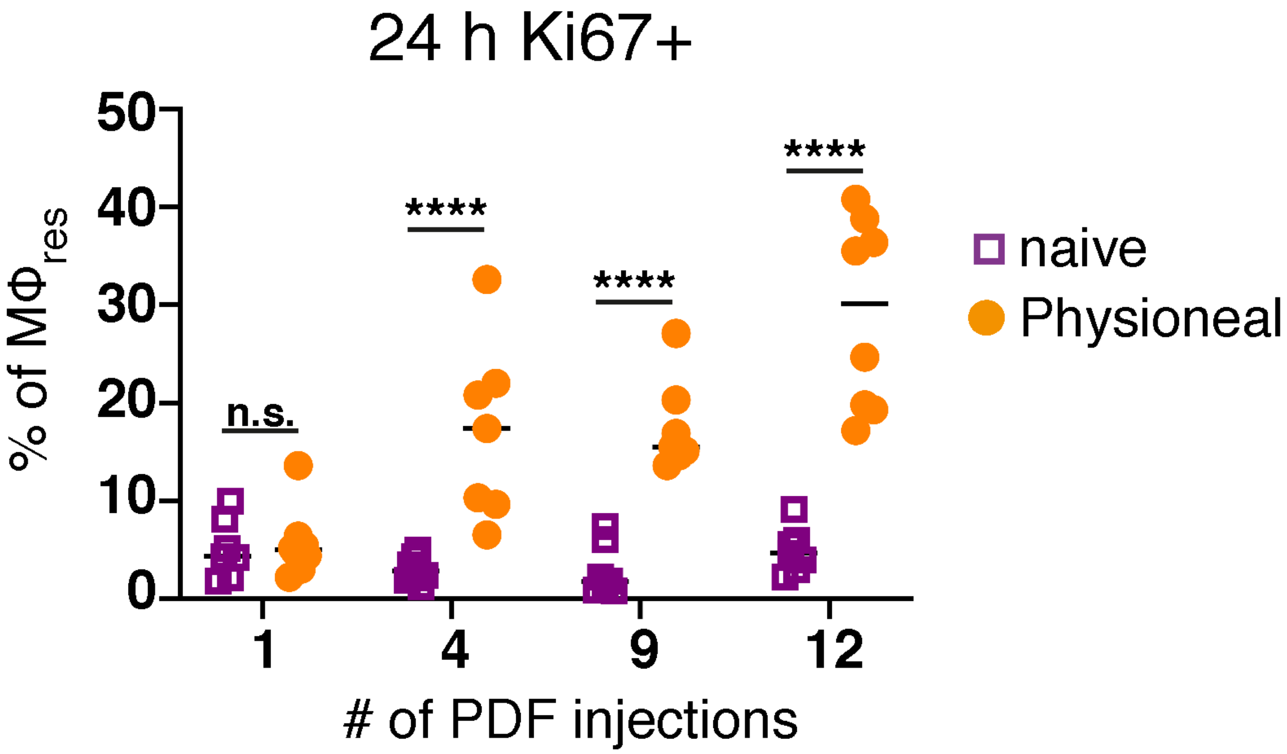

Figure S3: MΦres re-populate the peritoneal cavity through local proliferation following PD fluid injection.

C57BL/6 mice were injected with Physioneal (orange circle) or left untreated (purple square) 5 times a week for the indicated number of injections. At each timepoint whole PEC were isolated 24 h after the last injection and analysed by flow cytometry for intracellular expression of Ki67.

Datapoints depict individual animals and lines indicate mean. Data from separately performed experiments for each timepoint. Data analysed using 2-way ANOVA followed by Sidak's multiple comparison test after transformation. n.s.: not significant; *: $p<0.05$; **: $p<0.01$; ****: $p<0.0001$

Supplementary Figure S4

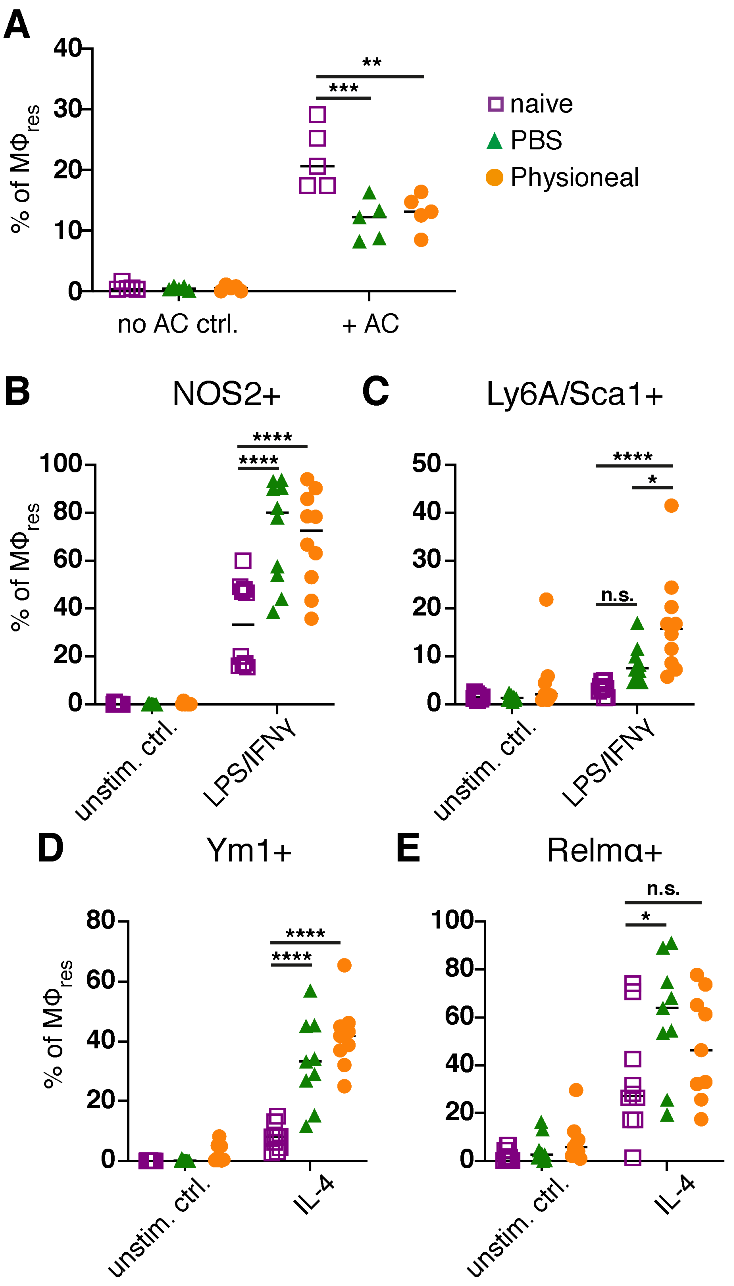

Figure S4: Altered MΦres phenotype is maintained after discontinuation of PD fluid injection.

C57BL/6 mice were injected 5 times a week for a total of nine injections with Physioneal (orange circles), sterile PBS (green triangles) or left untreated (purple squares). 24 h after the last injection whole PEC were isolated and stimulated in vitro with (A) pHRodo labelled apoptotic cells for 90 minutes, (B) LPS/IFNγ for 6 h or (C) IL-4 for 24 h and analysed by flow cytometry.

Datapoints depict individual animals and lines indicate mean. Data pooled from two independent experiments. Data analysed using 2-way ANOVA followed by Tukey's multiple comparison test after transformation. n.s.: not significant; *: p<0.05; **: p<0.01; ****: p<0.0001; ####: P<0.0001

Supplementary Figure S5

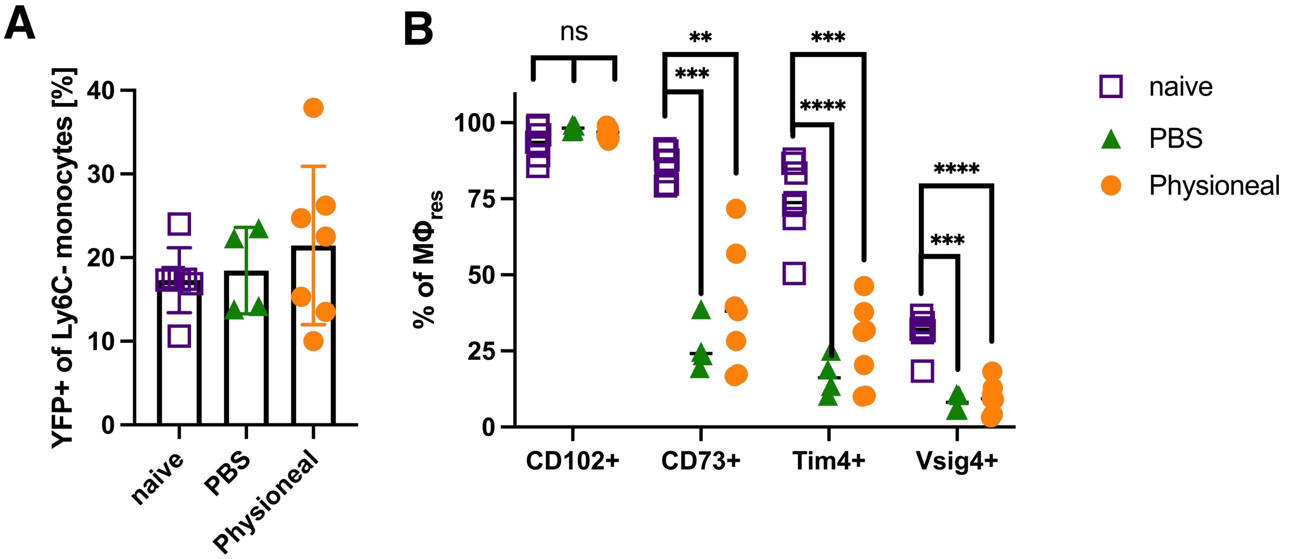

Figure S5: Cx3cr1[CreER]:R26-eyfp mice were injected daily with 5 mg tamoxifen for 5 consecutive days. Simultaneously, animals received daily injections, five times a week for a total of 9 injections of dialysis fluid (Physioneal, orange circles), PBS (green triangles) i.p. or were left untreated (naive, purple squares). A) Percent eYFP positive , Ly6C- monocytes (CD115+ CD19 -) in the blood.
B) Expression of CD102, CD73, Tim4 and Vsig4 on peritoneal $M\Phi_{res}$ in the mice analysed in A.
